# *Escherichia coli *inactivation kinetics in anaerobic digestion of dairy manure under moderate, mesophilic and thermophilic temperatures

**DOI:** 10.1186/2191-0855-1-18

**Published:** 2011-07-15

**Authors:** Pramod K Pandey, Michelle L Soupir

**Affiliations:** 1 Agricultural and Biosystems Engineering Department, Iowa State University, Ames, 50011, USA

**Keywords:** *E. coli*, inactivation kinetic, dairy manure, anaerobic digestion, temperature

## Abstract

Batch anaerobic digestion experiments using dairy manure as feedstocks were performed at moderate (25°C), mesophilic (37°C), and thermophilic (52.5°C) temperatures to understand *E. coli*, an indicator organism for pathogens, inactivation in dairy manure. Incubation periods at 25, 37, and 52.5°C, were 61, 41, and 28 days respectively. Results were used to develop models for predicting *E. coli *inactivation and survival in anaerobic digestion. For modeling we used the decay of *E. coli *at each temperature to calculate the first-order inactivation rate coefficients, and these rates were used to formulate the time - temperature - *E. coli *survival relationships. We found the inactivation rate coefficient at 52.5°C was 17 and 15 times larger than the inactivation rate coefficients at 25 and 37°C, respectively. Decimal reduction times (D_10_; time to achieve one log removal) at 25, 37, and 52.5°C, were 9 -10, 7 - 8 days, and < 1 day, respectively. The Arrhenius correlation between inactivation rate coefficients and temperatures over the range 25 -52.5°C was developed to understand the impacts of temperature on *E. coli *inactivation rate. Using this correlation, the time - temperature - *E. coli *survival relationships were derived. Besides *E. coli *inactivation, impacts of temperature on biogas production, methane content, pH change, ORP, and solid reduction were also studied. At higher temperatures, biogas production and methane content was greater than that at low temperatures. While at thermophilic temperature pH was increased, at mesophilic and moderate temperatures pH were reduced over the incubation period. These results can be used to understand pathogen inactivation during anaerobic digestion of dairy manure, and impacts of temperatures on performance of anaerobic digesters treating dairy manure.

## Introduction

In the United States, combined livestock production of cattle, swine, and sheep generates 49% of the total farm income, which is nearly 241 billion dollars (US Census Bureau 2010). The practice of applying livestock manure to recycle waste to enhance crop yield is more than 4,000 years old, and this practice continues to reduce farm fertilization costs in the developing as well as the developed world (WHO 2010; USEPA 2010). However, land application of manure can damage environmental ecosystems (USEPA, 2010) and create a risk to human health if not applied properly. Runoff from fields where manure has been applied can be a source of pathogen contamination in ambient water bodies including streams lakes and reservoirs and groundwater systems (Brennan et al. 2010; Gerba and Smith 2005; Mawdsley et al.1995; Pell 1997; Tyrrel and Quinton 2003; Unc and Goss 2004), particularly if a rainfall event occurs soon after application (Soupir et al. 2006).

Manure can contain numerous pathogenic organisms that are associated with human diseases including salmonella, *E. coli *O157:H7, *Yesinia*, *campylobacter*, *guardia*, and *cryptosporidium *(Klein et al. 2010; Létourneau et al. 2010; Massé et al. 2010; Ziemer et al. 2010). Treating manure before land application can greatly reduce the number of viable pathogens, and various methods such as drying (Amin-Nayyeri et al. 2010), composting (Maeda et al. 2010), heat treatment (Shepherd Jr et al. 2010), radiation treatment (Farag and Mohamed 1999; Sinton et al. 2007), aerobic digestion (Bortone 2009; Dumas et al. 2010; Shen et al. 2010), and anaerobic digestion (Aitken et al. 2005; Aitken et al. 2007; Lang and Smith 2008; Popat et al. 2010; Sung and Santha 2003; Wagner et al. 2009) are typically used. Of these, anaerobic digestion is crucial as it produces biogas, a source of energy, in addition to reducing pathogens.

The use of anaerobic digesters for biogas production and manure treatment is well established and has been implemented all over the world. A tremendous amount of research has been conducted on the anaerobic digestion process to enhance biogas production (Climent et al. 2007; He et al. 2008; Kim et al. 2010; Pandey et al. 2010; Yilmaz and Demirer 2008; Zaher et al. 2008), increase nutrient recovery (Banu et al. 2009; Carrere et al. 2010; Jin et al. 2009; Novak et al. 2010; Prapaspongsa et al. 2010; Wang et al. 2010), and reduce solids content (Forster-Carneiro et al. 2010; Gilroyed et al. 2010; Gong et al. 2010; Riau et al. 2010; Rubio-Loza and Noyola 2010).

Batch reactors are a useful tool to improve understanding of the outcomes of anaerobic digestion processes. Masse et al. (2010) evaluated efficiency of commercial-scale psychrophilic anaerobic digestion in sequencing batch reactors, operated at 7 or 14 days hydraulic retention time and 24°C. The authors found that the concentrations of fecal coliforms, *E. coli*, *Salmonella*, *Campylobacter spp*., and *Y. enterocolitica *were reduced to undetectable levels in pig manure. Côté et al. (2006) found nearly complete reduction of total coliforms and *E. coli *by anaerobic digestion at 20°C for 20 days while Baert et al. (2010) observed a 4 log decrease of Murine *Norovirus-1 *at 37 and 52°C after 13 and 7 days, respectively. Several studies have focused on pathogen inactivation in sludge anaerobic digestion at thermophilic temperatures to achieve Class A (complete pathogen inactivation) and Class B (incomplete pathogens removal) classification (Aitken et al. 2005; Popat et al. 2010; Puchajda et al. 2006; Smith et al. 2005; Wagner et al. 2009). For example, Shin et al. (2010) used the batch process to show decreases in bacteria 16S rRNA gene concentrations and organic removal efficiency in anaerobic digestion of secondary sludge, while Aitken et al. (2005) measured the inactivation of vaccine - strain poliovirus and eggs from helminth *Ascaris suum *at temperatures from 49 to 55°C in biosolids. Popat et al. (2010) used the batch process to estimate the inactivation kinetics of *Ascaris suum *and poliovirus type 1 (PVS -1) at temperatures ranging from 51 to 56°C. Despite previous studies to determine pathogen decay under anaerobic conditions, no information exists on the performance of anaerobic digestion in reducing pathogen concentrations in dairy manure.

Aitken et al. (2005) and Popat et al. (2010) provided important information about pathogen inactivation kinetics in anaerobic digestion. However, both of these studies are focused on anaerobic digestion of sludge obtained from municipal waste treatment facilities, and inactivation kinetics were determined at temperatures ranging from 49 to 56°C (thermophilic). Both studies emphasized the need for improving EPA's time - temperature relationships. Our objective is to determine the *E. coli *inactivation kinetics in dairy manure at moderate (25°C), mesophilic (37°C) and thermophilic (52.5°C) temperatures, and use the inactivation kinetics at these temperatures to derive the time - temperature - survival relationship for calculating *E. coli *survival in anaerobic digesters treating dairy manure.

## Materials and methods

### Anaerobic reactor setup

Fresh manure was collected from Iowa State University's dairy facility 24 hours prior to the start of the experiment to prepare the feedstock for the anaerobic reactors. To prepare the feedstock, 0.498 kg of fresh manure was mixed thoroughly in 1,500 ml of distilled water. Fibers and large solid particles in the manure were removed using a sieve with an 850 μm opening (USA standard testing sieve, No 20, Fisher Scientific Company).

Experiments were conducted for moderate temperature, mesophilic temperature, and thermophilic temperature at 25, 37, and 52.5°C, respectively. The moderate and mesophilic temperature experiments lasted 61 and 41 days respectively. Thermophilic experiments lasted for 28 days; however, *E. coli *was not detected after the fourth day of incubation. *E. coli *concentration was enumerated regularly; every 24 hours during the first two weeks for the moderate and mesophilic temperature experiments, and every 12 hours for the thermophilic experiment.

Each experiment included six anaerobic batch reactors, 250 ml serum bottles (Scientific Instrument Services, NJ, US), for incubating the feedstock. One hundred fifty ml of feedstock was transferred into each reactor, and then the reactors were sealed with a rubber septum (Sigma-Aldrich, sleeve stopper, MW 09194, St. Louis, MO, US). Before starting each experiment, an anaerobic environment at the reactor's headspace were created. The reactors were placed in an orbital water bath shaker (New Brunswick Scientific, Classic Series C7, 400768741, Edison, New Jersey, USA). During each experiment, the water bath shaker speed was maintained at 150 rpm. For analyzing *E. coli*, we collected incubated liquid slurry from three out of six reactors (randomly selected) using a 35 ml gas tight glass syringe (Micro - Mate, Popper & Sons Inc, New Hyde Park, NY). The biogas at each reactor was measured using a 35 ml gas tight glass syringe (Micro - Mate, Popper & Sons Inc, New Hyde Park, NY). To measure the biogas, the needle of the syringe was inserted into the septum, and the gas pressure in the bottle displaced the syringe plunger. The displaced volume indicated the amount of biogas produced, which was analyzed for CH_4 _content.

### *E. coli *enumeration

To enumerate *E. coli *concentrations in the incubated slurry, we used membrane filtration techniques using standard modified mTEC agar (growth media) (APHA, 1999). The liquid samples collected from the reactors were stored at 4°C immediately. To enumerate *E. coli *numbers, sample processing was performed within 24 hours of sample collection. The samples were serially diluted, and the diluted samples were filtered through 0.45 μm membrane filters (Millipore, FOEA 22910, HAWG047S, France). Membrane filters placed in petri dish with growth media were incubated at 44.5 ± 0.2°C for 24 hours. The red or magenta *E. coli *colonies grown in petri dish after incubation were enumerated. All analysis was performed in triplicate. Besides *E. coli *enumeration, we tested total solids (TS), volatile solids (VS), total nitrogen (TN), total phosphorous (TP), and total organic carbon (TOC) using standard methods (APHA, 1999).

### Data analysis

*E. coli *concentrations were averaged among the three replicates and used to estimate the first order kinetics:(1)

where *C_0 _*is the initial *E. coli *concentration (CFU/ml), *C *is the concentration (CFU/ml) at time *t*, *k *is first order inactivation rate coefficient (1/day) and *t *is time (day). The value of *k *(slope of regression) was estimated by liner regression between ln of *E. coli *concentration (CFU/ml) and time (day).

The effect of temperatures (moderate, mesophilic, and thermophilic) on the inactivation rate coefficient was estimated by the Arrhenius equation.(2)

where *k(T) *is the inactivation rate coefficient as a function of temperature for a given temperature and *A *is a pre-exponential factor, a constant for a given reaction. The *E_a _*is the activation energy (Joules/mol). The activation energy describes the influence of temperature on the magnitude of the first order reaction rate constant. The *R *is the gas constant (8.314472 Joules/mol K) and *T *is temperature (K). The *Ea/R *(slope of the regression line) and *A *(y-intercept) of equation 2 were obtained by plotting *ln k(T) *and *1/T*. Decimal reduction time (D_10_), time required to inactivate 90% of *E. coli*, was calculated at each temperatures using log of *E. coli *(CFU/ml) (Murphy 2002).

## Results

**Figure 1:**
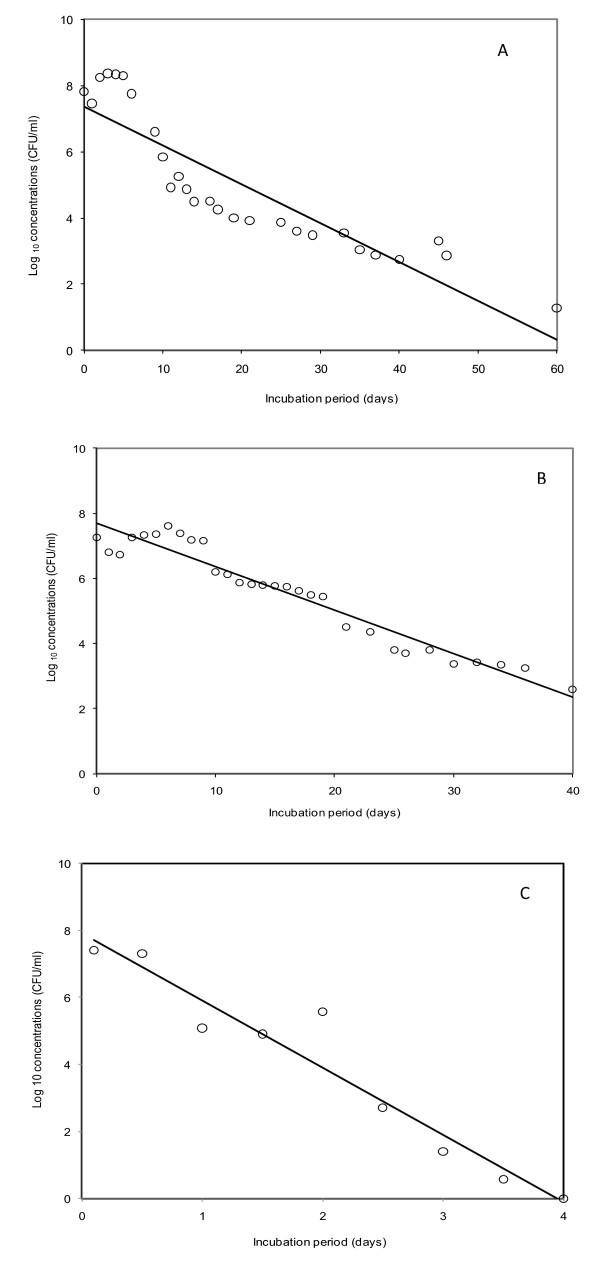
**Variations in *E. coli *concentrations with incubation periods**: (A) 25°C; (B) 37°C; (C) 52.5°C. The plot shows Log_10 _of *E. coli *concentrations (hollow circle) and regression line (solid black).

Results of *E. coli *inactivation at 25 (moderate), 37 (mesophilic), and 52.5°C (thermophilic) temperatures are shown in Figures 1a, b, and 1c, respectively. *E. coli *concentrations are plotted as logarithmic values to easily observe reduction over time. The inactivation was relatively high at 52.5°C, with inactivation rate coefficients 17 and 15 times greater than that of at 25 and 37°C experiments, respectively. At 25°C, greater than a six log reduction was observed during the 60 day incubation period; while, at 37°C approximately a six log reduction was obtained during 41 days of incubation. At 52.5°C greater than seven log reductions was obtained in 3.5 days; at this temperature after 4 days, *E. coli *reached to undetectable levels.

The characteristics of feedstock used in anaerobic digestion are shown in Table 1. The initial *E. coli *concentrations in feed stocks were 6.5 × 10^7^, 1.85 × 10^7^, 2.5 × 10^7 ^CFU/ml for the experiment at 25, 37, and 52. 5°C, respectively. Total organic content (TOC) for these feedstocks were 4500, 4170, and 3900 mg/L, respectively. While total nitrogen (TN) concentrations were 489, 390, 522 mg/L, total phosphorous concentrations were 153, 333, and 243 mg/L, respectively (Table 1). The inactivation rate constants, *R^2 ^*of linear regression, *T_c _*(time required for complete inactivation, and decimal reduction time (D_10_) are shown in Table 2. *E. coli *survived the longest in moderate temperature (> 60 days), and decayed quickly under thermophilic conditions (< 4 days). Compared to the moderate temperature experiment (*R^2 ^*= 0.81), better results were achieved for mesophilic and thermophilic experiments (R^2 ^> 0.90). The *T_c _*at moderate and mesophilic temperatures were greater than 60 and 40 days, respectively; the *T_c _*at thermophilic temperature was less than 4 days (Table 2). During the first 10 days at moderate temperature, *E. coli *inactivation trend deviated from the expected first-order kinetics, which was relatively consistent after day 10 of incubation.

**Table 1:** Initial feedstock characteristics

Temperature	TS (%)	VS (%)	pH	TN (mg/l)	TP (mg/l)	TOC (mg/l)	*E. coli (CFU/ml)*
Moderate (25°C)	1.39	1.12	7.52	489	153	4500	6.5 × 10^7^
Mesophilic (37°C)	2.05	1.25	7.40	390	333	4170	1.85 × 10^7^
Thermophilic (52.5°C)	1.75	1.28	7.55	522	243	3900	2.5 × 10^7^

**Table 2:** Summary of inactivation kinetics

Temperature	*k *(d^-1^)	^a^*T_c _*(days)	^b^*R^2^*	^**c**^***D****_**10**_***(days)**
Low temp (25°C)	0.1177	> 60	0.81	9 - 10
Mesophilic temp (37°C)	0.1335	> 40	0.94	7 - 8
Thermophilic temp (52.5°C)	2.0069	< 4	0.93	< 1

^a ^*T_c _*is required time for "complete" inactivation (first sample in which *E. coli *concentration was below detection limit.

^b^*R^2 ^*values were obtained from liner regression between Log_10 _*E. coli *concentrations (CFU/ml) and incubation periods (days).

^c^*D_10 _*decimal reduction time (reduction of the population by one log-unit) corresponding to Figures 1a, 1b, and 1c.

**Figure 2:**
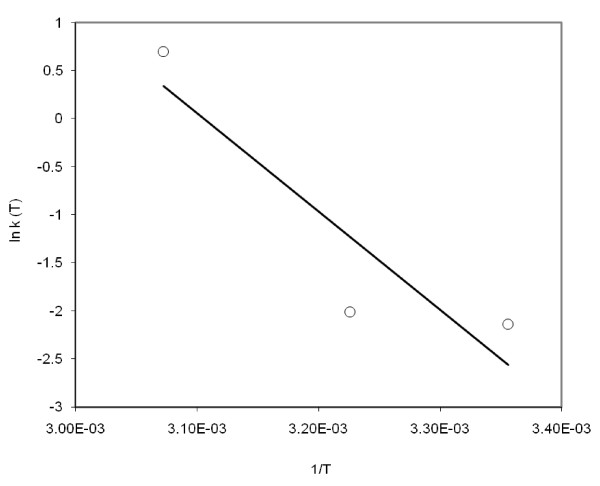
**Arrhenius plot for *E. coli *inactivation over the temperatures 25, 37 and 52.5°C**. The solid line indicates regression line, and hollow circles are inactivation rates of *E. coli*.

To model the impacts of temperature on the inactivation rate coefficients, we used the Arrhenius equation. Figure 2 shows the plot between *ln k(T) *and *1/T*. The *R^2 ^*of linear regression between *ln k (T) *and *1/T *was 0.82. The slope of the fitted line corresponds to activation energy of 84.9 kJ mol^-1^.

**Figure 3:**
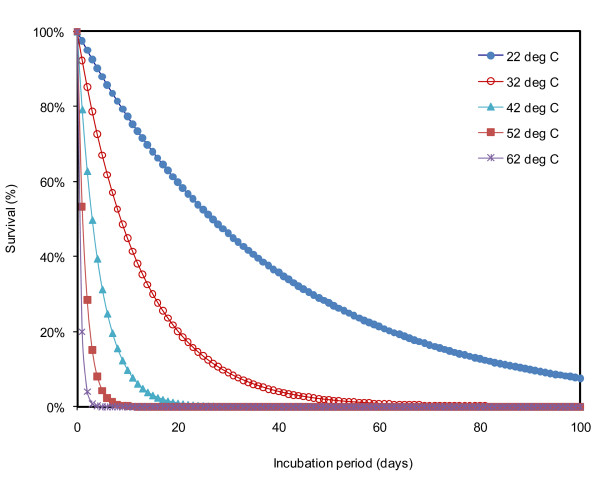
**Survival percentages of *E. coli *over incubation period for temperature range 22 - 62°C**. The blue solid circles indicates survival percentage at 22°C, red hollow circles is survival at 32°C, aqua solid triangles is survival at 42°C, red square at 52°C, and purple crosses are survival at 62°C.

Figure 3 shows *E. coli *survival with time for temperatures ranging from 22 - 62°C. The value of *A *in the Equation 2 was adjusted (we used A = 2.83 × 10^13^) until the predicted survival percentages at 25, 37, and 52.5°C were comparable to the measured survival percentages at these temperatures. A similar approach has been used previously by Aitken et al. (2005). Figure 3 shows that at 22°C on Day 60, survival was about 21.33%, while at 32°C, survival was reduced to less than 1%. Relatively, very fast inactivation was observed at 62°C; the survival reached 0.16% on Day 4.

Using the inactivation kinetics we derived the temperature - time - survival relationship as follows.(3)

where s is survival (%); *a *is constant (= 2.83 × 10^13^); *t *is the incubation time (day); and *T *is temperature (K). Further solving Equation 3 provided an equation for *t*.(4)

Equation 4 can be used to calculate a required time for a given *s *and *T*. It can be used to determine the required time for *E. coli *inactivation in anaerobic digesters treating dairy manure depending on temperature. In this study, we used filtered dairy manure for experiments, which may result in slightly different inactivation kinetics compared to unfiltered manure because of fiber and large solid manure particle presence. Those slight changes can be accounted for by adjusting *A *in equation 2.

**Figure 4:**
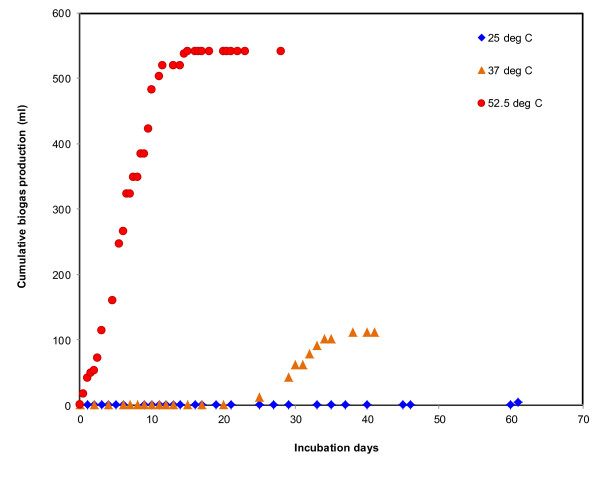
**Impacts of temperatures on biogas production**. The red solid circle indicates cumulative biogas production at 52.5°C, yellow triangles indicate biogas production at 37°C, and dark blue diamonds indicate biogas production at 25°C.

Descriptive statistics showing the impacts of temperature on total solid (TS), volatile solid (VS), pH, ORP, biogas production, and methane contents are shown in Table 3. Impacts of temperatures in biogas production are shown in Figure 4. At 52.5°C, biogas production was relatively very quick (within 14 hours of incubation), while at 37 and 25°C, onset of biogas was delayed until day 25 and 61, respectively. The cumulative biogas production at 37 and 25°C, were 111 and 11 ml over 41 and 61 days of incubation periods, respectively. The cumulative biogas production at 52.5°C (541.75 ml) was 4.8 and 49 times greater than that at 37, and 25°C. Methane content in biogas at 52.5°C varied between 44 and 70% with mean of 56 ± 18%, while at 37°C, it varied from 26 to 55% with mean of 40.6 ± 20.34 (Table 3). Methane content at 25°C was not measured as biogas production was very low.

**Table 3:** Changes in parameters over incubation period for different temperatures

Temperatures	Initial	Final	Changes (%)	Increase (+)/decrease (-)	Mean	Standard Deviation	^d^Range
Total solids (TS) (%)							

^a^25°C	1.39	0.99	29.06	-	1.37	0.18	0.99 - 1.67
^b^37°C	2.05	1.25	38.96	-	1.58	0.20	1.25 - 2.05
^c^52.5°C	1.75	0.82	53.20	-	1.43	0.38	0.79 - 2.12

Volatile solids (VS) (%)							

25°C	1.12	0.48	57.11	-	0.92	0.27	0.19 - 1.25
37°C	1.65	0.89	45.84	-	1.21	0.19	0.89 - 1.65
52.5°C	1.28	0.48	62.72	-	1.43	0.38	0.48 - 1.88

pH							

25°C	7.52	6.86	8.78	-	7.14	0.25	6.78 - 7.56
37°C	7.40	6.53	11.70	-	6.82	0.29	6.34 - 7.40
52.5°C	7.55	9.54	20.86	+	8.95	0.70	7.55 - 9.70

ORP (mV)							

25°C	-300	-90		+	-229	124	-353 - 14.70
37°C	-346	-150		+	-314	44	-360 to - 150
52.5°C	-288	-20		+	-111	101	-288 to -10

Biogas (ml); methane (%)							

25°C	0	0			0.34; ND	1.42; ND	0 - 5; ND
37°C	0	0			4.4; 40.6	8.1; 20.34	0 - 30; 26 - 55
52.5°C	0	0			18; 56	22; 18	0 - 87; 44 - 70

^a^incubation period = 61 days; ^b^incubation period = 41 days; ^c^incubation period = 28 days; ^b^Range = minimum - maximum; ND = not detected

**Figure 5:**
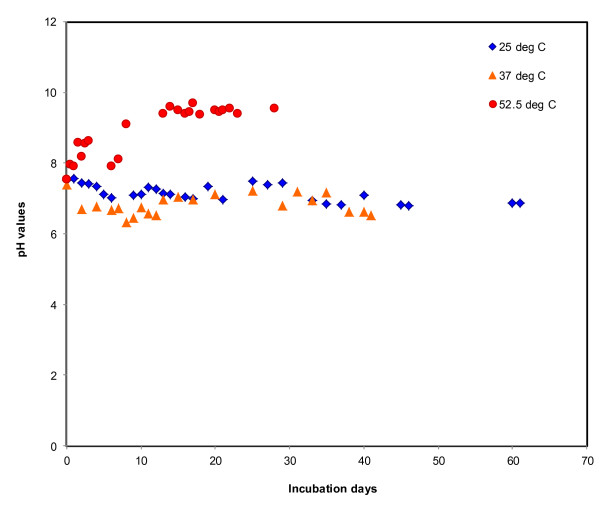
**Impacts of temperatures on pH changes**. The red solid red circle indicates pH changes at 52.5°C, yellow triangles indicate pH changes at 37°C, and dark blue diamonds indicate pH changes at 25°C.

The variation in pH over incubation period is shown in Figure 5. At 37 and 25°C, pH was reduced over time and both temperatures show similar patterns; however, at 52.5°C, pH was increased (Table 3). The mean pH over incubation periods at 52.5, 37, and 25°C, were 7.14 ± 0.25, 6.82 ± 0.29, and 8.95 ± 0.0.70, respectively. At 52.5°C, pH was increased about 20.86%, while at 37 and 25°C, pH was decreased 11.70 and 8.78%, respectively, at the end of incubation.

**Figure 6:**
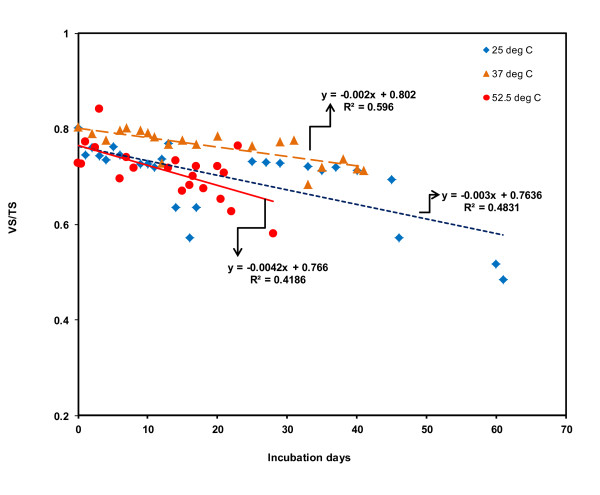
**Impacts of temperatures on changes in ratio between VS and TS (VS/TS)**. The red solid line indicate regression line, and red circle indicated VS/TS reductions at 52.5°C; the yellow long dash line indicates regression line, and yellow triangles indicate VS/TS at 37°C; the blue short dash line indicates regression line, and the blue diamond indicate VS/TS at 25°C.

The impacts of temperatures on variation in VS/TS are shown in Figure 6. The reduction in VS/TS at 52.5°C was higher than that at 37, and 25°C. The initial VS/TS ratio at 25, 37, and 52.5°C was 0.80, 0.80, and 0.73, respectively. The reductions in VS/TS over incubation period were 39, 11, and 20%, at 25, 37, and 52.5°C, respectively. The liner regression lines for VS/TS changes over incubation period are shown in Figure 6. The descriptive statistics of total solid (TS) and volatile solid (VS) is shown in Table 3. The TS and VS at 25°C was reduced approximately 29 and 57% at the end of incubation period. At 37°C, TS and VS reduction was 38 and 46%. At thermophilic temperature, TS and VS reduction was 53 and 63%. At the beginning of incubation, ORP values, which indicates the redox potential, were - 300, -346, -288 mV, at 25, 37, and 52.5°C, respectively. Over incubation period, ORP values varied from -353 to 14.70, -360 to -150, and -288 to - 10 mV, at 25, 37, 52.5°C, respectively. The descriptive statics of ORP changes are shown in Table 3.

## Discussion

Previous studies such as Aitken et al. (2005) and Popat et al. (2010) have used batch anaerobic digestion at thermophilic temperatures to understand temperatures' impact on pathogen inactivation and provided important information on biosolids' virus inactivation. The studies reported that inactivation rate increases with increasing temperature; however their studies were focused on thermophilic temperatures only. Our study focuses on understanding *E. coli *inactivation in dairy manure anaerobic digestion at a wide range of temperatures (22 - 62°C; our results of *E. coli *inactivation are based on three different ranges of temperatures (moderate, mesophilic, and thermophilic).

As shown in Figure 1a and 1b, the growths of *E. coli *was observed during initial phases. Initial growth spikes have also been reported by Smith et al. (2005) who studied the decay of *E. coli *NCTC 9001, *E. coli *O148 and *E. coli *O158 inoculated in 1.8 ml tubes of culture broth. Smith et al. (2005) observed spikes in *E. coli *concentrations at 37°C, but not at 55°C. Aitken et al. (2005) and Popat et al. (2010) have studied initial temperature perturbation influence extensively. Initial perturbation occurs during inoculation, when a volume of liquid at a given temperature is inoculated with a volume of cooler liquid. Nolf (1932) reported a required time of 2 -3 minutes for the temperature of incubation mixture to reach the temperature of the water bath. In this study, for the moderate temperature experiment, the water temperature was unchanged, at mesophilic temperature, the water temperature went slightly below 37°C for about 2 minutes, and at thermophilic temperature, water temperature went slightly below 52.5°C for less than 2 minutes. We ignored these minor temperature variations in our analysis because the period of initial temperature perturbation was minimal compared to the total incubation period (65, 45, and 29 days for moderate, mesophilic, and thermophilic, respectively).

Popat et al. (2010) reported inactivation energy of viruses of biosolids. For comparing our results, we were not able to find published reports; that have estimated inactivation energy of *E. coli *in dairy manure. Compared to Popat's study, who calculated inactivation energies of *Ascaris suum *and Poliovirus as of 105 and 39 kJ mol^-1^, respectively; our results are different. Another study by Aitken et al. (2005), have estimated inactivation energies of 580 and 550 kJ mol^-1 ^for *Ascaris suum *and Poliovirus, respectively. Both studies estimated the inactivation energies in the thermophilic temperature range (49 - 56°C); however, the differences in estimated inactivation energy is relatively large. The possible reason could be the differences in the sludge material used for incubations. While Aitiken et al. (2005) reported > 3 log reduction of *Ascaris *within 30 minutes of incubation, Popat et al. (2010) reported two hours for the same reduction. Both studies speculated that such high activation energies are characteristics of protein denaturation, and therefore the primary inactivation mechanism at thermophilic temperatures could be capsid protein denaturation. The potential cause for the low inactivation energy values in our study could be either differences in types of measured pathogens or feedstock characteristics.

The effects of temperatures on *E. coli *inactivation are apparent (shown in Figures 1, 2, 3), however, inactivation can also be influenced by other factors such as ammonia, pH, feedstock characteristics, volatile fatty acids, protein, fats, and carbohydrates. The impacts of these other factors in inactivation kinetics are unclear (Aitken et al. 2007; Popat et al. 2010). For sludge digestion it has been reported that the increased concentration of ammonia increases the inactivation of pathogenic viruses (Pecson et al. 2007; Ward and Ashley 1977). The presence of protective agents such as anionic detergents in sludge, reduces the inactivation (Ward and Ashley 1978). Volatile fatty acids, which are produced by acidogenic bacteria during anaerobic digestion (Pandey et al. 2010), enhance the inactivation rate (Popat et al. 2010). Collagen, a main protein of connective tissues in animals, significantly reduces the rate of virus inactivation (Milo 1971). Pecson et al. (2007) stated that pH effects are not significant; however, the authors have also reported that pH influence cannot be separated from ammonia concentration and temperature, and the temperature effects are dominant.

The initial characteristics of the feedstock often differ among treatment facilities, and therefore, the reported inactivation kinetics should be applied with caution. For example, Aitken et al. (2007) studied the inactivation of putative pathogenic *E. coli *O157:H7 and putative non - pathogenic *E. coli *in dairy cattle manure, and reported a mean inactivation coefficient, *k*, of 0.25 min^-1 ^and 0.23 min^-1 ^for pathogenic and non-pathogenic *E. coli*, respectively at 55°C; and 0.034 min^-1 ^and 0.018 min^-1^, respectively at 50°C. In our study, we report an inactivation coefficient of 2.01 day^-1 ^(0.00139 min^-^1) at 52.5°C for mixed wild strains of *E. coli*, which is significantly less. The possible reason for this discrepancy could be differences in the feedstock characteristics used for incubation or the heat resistance capacity of the *E. coli *strains used by Aitken et al. (2007).

Both experimental methods and tested pathogenic organisms can influence the results. Often the inactivation coefficients of pathogens are estimated in laboratories using either aqueous medium or biosolids or manure inoculated with pathogens. For example, inactivation kinetics calculated by Aitken et al. (2007), Aitken et al. (2005), and Popat et al. (2010); theses studies have determined inactivation kinetics by spiking a known concentration of pathogens into the feedstock, prior to anaerobic digestion. In our study, we enumerated the native *E. coli *which were already present in dairy manure. The results can be more reliable, when native organisms are used in experiments. We tested *E. coli *because it is a popular indicator among researchers and recommended by the EPA (USEPA 2010) for detecting the presence of faecal contamination and viruses in waters (Grisey et al. 2010). Compared to reported time - temperature relationships used by the EPA, which calculates pathogen inactivation by extrapolating between two temperatures (Popat et al., 2010), we used three different temperatures (moderate, mesophilic, and thermophilic) to calculate pathogen inactivation kinetics. Aitken et al. (2005) and Popat et al. (2010) have proposed to improving the EPA's time-temperature relationship as it neglects temperature mediated changes in deactivation mechanisms. The relationship we developed to estimate *E. coli *inactivation includes the temperature-mediated changes.

Besides impacts on *E. coli *inactivation, temperatures also influences changes in parameters such as pH, TS, VS, ORP, biogas production, and methane contents. As shown in Table 3 and Figure 4, biogas production at thermophilic temperature was relatively very quick and high compared to mesophilic and moderate temperatures. This indicates that the anaerobic process was enhanced with increased temperatures, and thermophilic temperatures can be the best for increasing biogas production as well as for *E. coli *reduction. The methane content in biogas was also higher at thermophilic temperatures compared to moderate and mesophilic temperatures. Relatively higher VS and TS reduction were observed at thermophilic temperature within 28 days of incubation compared to 61 and 41 days of incubation at 25 and 37°C. Other parameters, for example, ORP values indicated  better reactor performance at the thermophilic temperature. At the higher temperature, negative ORP values were coincided with improved anaerobic digester performance; similar results, for example, negative ORP values with improved anaerobic digestion of food waste are reported by Kuo and Lai (2010). The high ORP indicates low redox potential, which can cause the low biogas production; methanogens, which produce biogas, are reported to be relatively more active at low ORP values.

In summary, we used moderate, mesophilic, and thermophilic temperatures to understand *E. coli *inactivation in anaerobic digestion of dairy manure. Besides *E. coli *inactivation, we also studied the impacts of temperatures on other parameters including, pH, TS, VS, ORP, biogas production, and methane content. Results indicated that *E. coli *inactivation and biogas production greatly depend on temperatures and incubation period. At thermophilic temperature, *E. coli *inactivation and biogas production were faster than that of at moderate and mesophilic temperatures. We used this information to develop the model for predicting *E. coli *inactivation in anaerobic digestion of dairy manure and for developing the time -temperature - *E. coli *survival relationships. The relationships we proposed in Equations 3 and 4 can be used to understand *E. coli *inactivation in anaerobic digesters treating dairy manure.

## Competing interests

The authors declare that they have no competing interests.
